# Investigation of Cytotoxicity, Apoptosis, and Oxidative Stress Response of Fe_3_O_4_-RGO Nanocomposites in Human Liver HepG2 cells

**DOI:** 10.3390/ma13030660

**Published:** 2020-02-02

**Authors:** Maqusood Ahamed, Mohd Javed Akhtar, M. A. Majeed Khan

**Affiliations:** King Abdullah Institute for Nanotechnology, King Saud University, Riyadh 11451, Saudi Arabia; mohd.j.akhtar@gmail.com (M.J.A.); mmkhan@ksu.edu.sa (M.A.M.K.)

**Keywords:** RGO, Fe_3_O_4_-RGO nanocomposites, biocompatibility, cytotoxicity, biomedical application

## Abstract

Iron oxide–reduced graphene oxide (Fe_3_O_4_-RGO) nanocomposites have attracted enormous interest in the biomedical field. However, studies on biological response of Fe_3_O_4_-RGO nanocomposites at the cellular and molecular level are scarce. This study was designed to synthesize, characterize, and explore the cytotoxicity of Fe_3_O_4_-RGO nanocomposites in human liver (HepG2) cells. Potential mechanisms of cytotoxicity of Fe_3_O_4_-RGO nanocomposites were further explored through oxidative stress. Prepared samples were characterized by UV-visible spectrophotometer, X-ray diffraction, scanning electron microscopy, transmission electron microscopy, and energy dispersive spectroscopy. The results demonstrated that RGO induce dose-dependent cytotoxicity in HepG2 cells. However, Fe_3_O_4_-RGO nanocomposites were not toxic. We further noted that RGO induce apoptosis in HepG2 cells, as evidenced by mitochondrial membrane potential loss, higher caspase-3 enzyme activity, and cell cycle arrest. On the other hand, Fe_3_O_4_-RGO nanocomposites did not alter these apoptotic parameters. Moreover, we observed that RGO increases intracellular reactive oxygen species and hydrogen peroxide while decrease antioxidant glutathione. Again, Fe_3_O_4_-RGO nanocomposites did not exert oxidative stress. Altogether, we found that RGO significantly induced cytotoxicity, apoptosis and oxidative stress. However, Fe_3_O_4_-RGO nanocomposites showed good biocompatibility to HepG2 cells. This study warrants further research to investigate the biological response of Fe_3_O_4_-RGO nanocomposites at the gene and molecular level.

## 1. Introduction

Graphene is a single layer of carbon atoms in a tightly packed two-dimensional honeycomb lattice with unique structural, optoelectronic, thermal, and mechanical characteristics [1,2]. Due to unique physicochemical properties, graphene has shown great potential for various applications in fields such as energy and biomedicine [3,4]. However, poor solubility of graphene in physiological media hindered its application in the biomedical field. Graphene derivatives such as graphene oxide (GO) and reduced graphene oxide (RGO) has now received great attention due to their excellent solubility in physiological media, good biocompatibility at real human exposure level, cost effective production and ability to integrate with other nanomaterials [5,6]. The GO and RGO contains a large number of residual oxygen functional groups with a large number of surface defects. The oxygen functional groups and surface defects are very reactive and can be utilized in developing advanced GO/RGO-based nanocomposites that are useful in numerous applications including imaging, targeted drug delivery, and cancer therapy [7,8].

The integration of inorganic nanoparticles (NPs) with GO/RGO to form nanocomposites has become a hot topic of current research because of their superior properties that cannot be achieved by either component alone. Generally, it is believed that the anchoring of inorganic NPs onto GO/RGO sheets may prevent the restacking of sheets and enhance their physicochemical properties [9]. In addition, to keep the surface area to volume ratio high, which is required for biomedical applications, incorporation of inorganic NPs on GO/RGO sheets is very important [10]. Recently, Gurunathan et al. [11] reported that GO-platinum nanocomposite induces cytotoxicity and genotoxicity in human prostate cancer (LNCaP) cells. The GO-silver nanocomposites enhanced the anticancer potential of salinomycin against ovarian cancer stem cells (OvCSCs) [12].

Superparamagnetic Fe_3_O_4_ NPs emerged as an excellent candidate for biomedical applications because of their chemical stability, low toxicity and ease of functionalization with other nanomaterials [13,14]. Various nanocomposites containing Fe_3_O_4_ NPs in thermo-sensitive polymeric micelles, liposomes, and lipid NPs loading anticancer drugs for targeted delivery have been reported [15,16]. Researchers are now investigating the potential of Fe_3_O_4_-RGO nanocomposites for their application in environmental remediation, lithium ion batteries, and biomedicine [4,10,17]. Recent studies also suggested that Fe_3_O_4_-RGO is a suitable multifunctional nanocomposite for magnetic-targeted drug delivery and magnetic resonance imaging (MRI) [18,19]. Therefore, it is necessary to assess the biocompatibility/toxicity of Fe_3_O_4_-RGO nanocomposites before their wide-spread application. Information on the biocompatibility/toxicity of Fe_3_O_4_-RGO nanocomposites at cellular and molecular level is scarce. This study was designed to synthesize, characterize, and assess the cytotoxicity and apoptosis response of Fe_3_O_4_-RGO nanocomposites in human liver (HepG2) cells. Potential mechanisms of cytotoxicity of Fe_3_O_4_-RGO nanocomposites were further explored through reactive oxygen species (ROS) generation and oxidative stress. We also prepared RGO nanosheets to compare their toxicity with Fe_3_O_4_-RGO nanocomposites. The HepG2 cell line is a human hepatic model that has been widely used in nanotoxicity and nanomedicine research [20,21]. The HepG2 cell line was chosen in this study because several animal model studies demonstrated that the liver is one of the target organs of GO and RGO after intravenous administration [22,23].

## 2. Materials and Methods 

### 2.1. Synthesis of RGO, Fe_3_O_4_ NPs and Fe_3_O_4_-RGO Nanocomposites

Graphene oxide (GO) was prepared from graphite powder using a modified Hummers’ method [24,25]. Reduced graphene oxide (RGO) was prepared by the chemical reduction of GO using hydrazine hydrate. The Fe_3_O_4_-RGO nanocomposites were synthesized by a chemical co-precipitation procedure. Briefly, 50 mg of GO powder were suspended in 100 mL of deionized water by ultra-sonication for 30 min, followed by the addition of 0.2 g of FeCl_3_.6H_2_O and 0.1 g of FeCl_2_.4H_2_O. Then, reaction mixture was purged with N2 gas to remove dissolved O_2_ and stirred for 1 h. Ammonium hydroxide (15 mL of 8M NH_4_OH) aqueous solution was mixed drop wise to precipitate ferrous and ferric ions. Hydrazine hydrate (1 mL of 70% w/w) was further added to the mixture, and the reaction was carried out at 60 °C for 2 h under magnetic stirring. The Fe_3_O_4_-RGO product was collected by magnetic separation, washed several times with water/ethanol, and dried under vacuum at 60 °C. Bare Fe_3_O_4_ NPs were also synthesized by the same method without adding GO.

### 2.2. Characterization

Optical absorption spectra of Fe_3_O_4_ NPs and Fe_3_O_4_-RGO nanocomposites were measured between 300–900 nm wavelengths using UV-visible spectrophotometer (Shimadzu, Columbia, MD, USA). X-ray diffractometer (XRD, PanAnalytic X’Pert Pro) (Malvern, WR14 1XZ, United Kingdom) with Cu-Kα radiation (λ = 0.15405 nm, at 45 kV and 40 mA) was employed to examine the phase purity and crystallinity of prepared RGO, Fe_3_O_4_ NPs, and Fe_3_O_4_-RGO nanocomposites. Structural characterization of RGO, Fe_3_O_4_ NPs, and Fe_3_O_4_-RGO nanocomposites were further assessed by scanning electron microscopy (SEM, JSM-7600F, JEOL, Inc., Tokyo, Japan) and transmission electron microscopy (TEM, JEM-2100F, JEOL, Inc., Tokyo, Japan). Elemental composition of Fe_3_O_4_-RGO nanocomposites were evaluated by energy dispersive spectroscopy (EDS) (JEOL, Inc., Tokyo, Japan) associated with TEM.

### 2.3. Cell Culture and Exposure of NPs and Nanocomposites

Human liver (HepG2) cells were cultured in Dulbecco’s modified eagle’s medium (DMEM) (Invitrogen, CA, USA) containing 10% fetal bovine serum (Invitrogen), 100 U/mL penicillin, and 100 µg/mL streptomycin (Invitrogen) with the supply of 5% CO_2_ at 37 °C. At 75%–85% confluence, cells were harvested and further cultured for biochemical studies. 

Cells were allowed for 24 h to attach on surface of culture plate before exposure of NPs and nanocomposites. Dry powder of NPs and nanocomposites were suspended in DMEM at a concentration of 1 mg/mL. Stock solution was further diluted to different concentrations required for cytotoxicity experiments. The various concentrations of NPs and nanocomposites were sonicated at room temperature for 30 min at 40 W to avoid agglomeration before exposure to cells. Cells not exposed to NPs or nanocomposites served as control for each experiment. 

### 2.4. Biochemical Studies

The HepG2 cells were treated with different concentrations (1, 5, 10, 25, 50, 100 and 200 µg/mL) of Fe_3_O_4_ NPs, RGO and Fe_3_O_4_-RGO nanocomposites for 24 h. Cell viability was assessed by MTT assay [26]. Live cells have the ability to reduce MTT in blue formazon product dissolved in a solvent, and absorbance was recorded at 570 nm employing a microplate reader (Synergy-HT, Biotek, Vinnoski, VT, USA). Based on the MTT cell viability results, we chose one concentration (100 µg/mL) of each material for further experiments.

LDH enzyme leakage was assayed using a BioVision kit (BioVision, Milpitas, CA, USA) and detail procedures were explained in our previous work [27]. Cationic fluorochrome rhodamine-123 (Rh-123) was used to examine the mitochondrial membrane potential (MMP) [28]. The Rh-123 binds to the mitochondria of living cells in a membrane potential-dependent manner. MMP level was estimated by two distinct procedures—qualitative examination by a fluorescent microscope (DMi8, Leica Microsystems, Wetzlar, Germany) and quantitative assay by a microplate reader (Synergy-HT, Biotek, Vinnoski, VT, USA). Activity of caspase-3 enzyme was assayed using a commercial kit (BioVision, Milpitas, CA, USA). A propidium iodide (PI) probe was used to assess the cell cycle phases using a Flow cytometer (Coulter Epics XL/XI-MCL) (Beckman, Ramsey, MN, USA) via FL4 filter (585 nm) [28]. Intracellular reactive oxygen species (ROS) level was assessed using dichlorofluorescin diacetate (DCFH-DA) probe as described in our previous work [26]. DCFH-DA passively enters the cells and reacts with ROS to form a fluorescent compound called dichlorofluorescein (DCF). Fluorescent intensity of DCF was determined by two different methods—qualitative analysis by a fluorescent microscope (DMi8, Leica Microsystems, Wetzlar, Germany) and quantitative assay by a microplate reader (Synergy-HT, Biotek, Vinnoski, VT, USA). Intracellular level of hydrogen peroxide (H_2_O_2_) was assayed using a commercial kit (Sigma-Aldrich, St. Louis, MO, USA). The intracellular level of glutathione (GSH) was measured following the protocol of Ellman [29] using 5,5-dithio-bis-nitrobenzoic acid (DTNB). Protein content was estimated by Bradford’s method [30].

### 2.5. Statistics

One-way analysis of variance (ANOVA) followed by Dunnett’s multiple comparison tests were used for statistical calculation of biochemical studies. The *p* < 0.05 was ascribed as statistically significance.

## 3. Results and Discussion

### 3.1. Characterization of RGO, Fe_3_O_4_ NPs, and Fe_3_O_4_-RGO Nanocomposites

**Figure 1:**
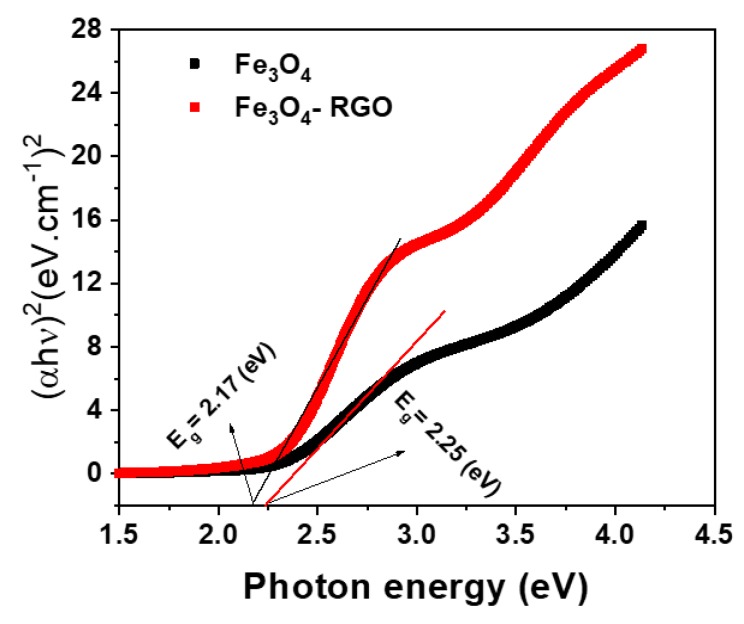
Optical characterization of Fe_3_O_4_ NPs and Fe_3_O_4_-RGO nanocomposites. Fe_3_O_4_: iron oxide, NPs: nanoparticles, RGO: reduced graphene oxide.

Optical characterization of Fe_3_O_4_ NPs and Fe_3_O_4_-RGO nanocomposites was assessed by UV-visible spectroscopy. It should be noted that the absorption edge was red shifted (absorption graph not given). Absorption coefficient (α) is measured utilizing the known relation α = 2.303A/x, where A is the absorbance and x is the cuvette’s thickness. Then, utilizing absorption coefficient and frequency (ν) of incident radiation. Band gap energy (Eg) was estimated by Tauc’s formula, αhν = B(hν-Eg)^n^, where h is the Plank’s constant, B is the constant and n is equal to 1/2 for the allowed direct optical transition. From the Tauc plot of (αhν)^2^ versus hν, the direct band gap energy (Eg) values were 2.25 eV and 2.17 eV for Fe_3_O_4_ NPs and Fe_3_O_4_-RGO nanocomposites, respectively (Figure 1). Our results were in agreements with other reports [31]. This phenomenon (decreasing of band gap) is useful for enhancing the light absorption of Fe_3_O_4_-RGO nanocompsoties that can be applied in the biomedical field.

**Figure 2:**
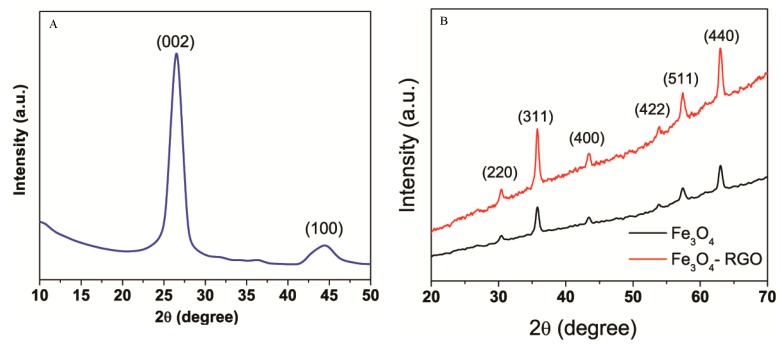
XRD characterization of RGO (**A**) and Fe_3_O_4_ NPs & Fe_3_O_4_-RGO nanocomposites (**B**). XRD: X-ray diffraction, RGO: reduced graphene oxide, Fe_3_O_4_: iron oxide, NPs: nanoparticles.

Crystal structure and purity of prepared samples (RGO, Fe_3_O_4_ NPs, and Fe_3_O_4_-RGO nanocomposites) were examined by XRD. The XRD spectra of RGO represents an intense reflection plane (002) at 2θ = 26.54 (Figure 2A). Presence of another diffraction peak at 2θ = 44.42, which is attributed to the (100) plane indicated the polycrystalline nature of RGO [31). Figure 2B presents the XRD spectra of bare Fe_3_O_4_ NPs and Fe_3_O_4_-RGO nanocomposites. All the diffraction peaks of both Fe_3_O_4_ NPs and Fe_3_O_4_-RGO nanocomposites were well indexed to the face center cubic (fcc) crystal structure of magnetite (JCPD No. 65-3107). The average particle size was calculated using Scherrer’s formula for the most intense diffraction peak (311). The average crystallite size of Fe_3_O_4_ NPs was estimated to be 14.8 nm for bare Fe_3_O_4_ and 12.3 for Fe_3_O_4_-RGO nanocomposites. No clear diffraction peak of RGO was observed in the XRD spectra of the Fe_3_O_4_-RGO nanocomposites, suggesting that homogeneous distribution of Fe_3_O_4_ NPs effectively inhibited the restacking of the RGO sheets [32].

**Figure 3:**
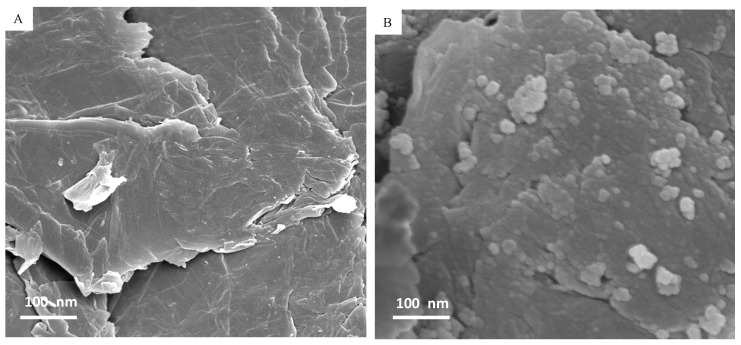
SEM characterization of RGO (**A**) and Fe_3_O_4_-RGO nanocomposites (**B**). SEM: scanning electron microscopy, RGO: reduced graphene oxide, Fe_3_O_4_: iron oxide.

**Figure 4:**
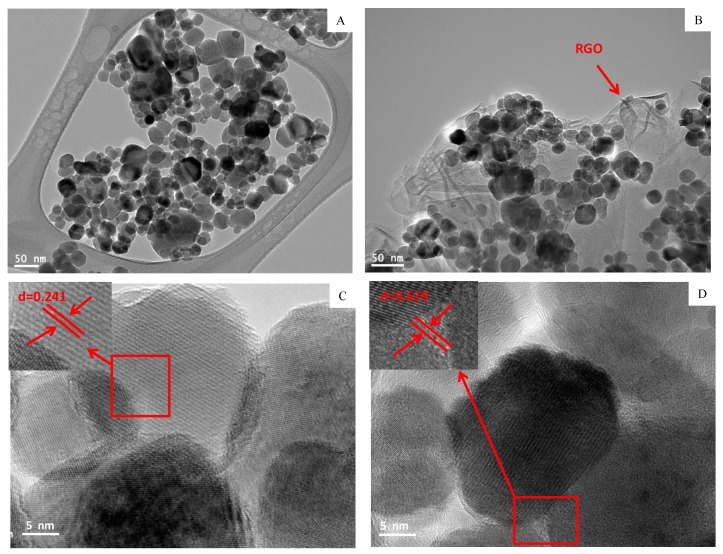
Low resolution TEM images (**A**,**B**) and high resolution TEM images of Fe_3_O_4_ NPs and Fe_3_O_4_-RGO nanocomposites (**C**,**D**). TEM: transmission electron microscopy, RGO: reduced graphene oxide, Fe_3_O_4_: iron oxide, NPs: nanoparticles.

Structural characterization of prepared samples was further done by SEM and TEM. The SEM image of RGO (Figure 3A) depicted the formation of a few layers of RGO with visible wrinkles and silky morphology due to high aspect ratio. The SEM micrograph of the Fe_3_O_4_-RGO nanocomposites (Figure 3B) suggested that Fe_3_O_4_ NPs were well anchored on the RGO sheets. The TEM image (Figure 4A) presents a nearly homogenous size distribution of almost spherical shaped Fe_3_O_4_ NPs. Figure 4B depicted that Fe_3_O_4_ NPs were tightly anchored on the surface of thin RGO sheets. Isolated Fe_3_O_4_ NPs were rarely observed in TEM study, indicating that most of the Fe_3_O_4_ NPs were attached on the surface of RGO sheets [33]. Besides, Fe_3_O_4_NPs on the surface of RGO may act as spacers to reduce the restacking of RGO sheets and to avoid the reduction of their high surface area [34]. The average size of Fe_3_O_4_ NPs in bare Fe_3_O_4_ and Fe_3_O_4_-RGO nanocomposites was around 15.6 and 13.8 nm, which was almost close to size calculated from XRD. The high resolution TEM images (Figure 4C,D) suggested high crystalline phase of NPs with lattice fringes of 0.241 and 0.619 nm, which corresponds to the d-spacing of the intense peaks (311) and (002) of face-centered cubic Fe_3_O_4_ NPs and Fe_3_O_4_-RGO nanocomposites [4]. The chemical composition of prepared Fe_3_O_4_-RGO nanocomposites was determined by EDS. The EDS spectra suggest that Fe, O, and C were main elements in Fe_3_O_4_-RGO nanocomposites. The peaks of Cu and C were observed due to the use of a carbon-coated copper TEM grid (Figure S1). Large surface area to volume ratio of prepared Fe_3_O_4_-RGO nanocomposite can be utilized in targeted drug delivery. Our characterization data of prepared Fe_3_O_4_-RGO nanocomposite were similar to other reports [31].

### 3.2. Cytotoxicity of Fe_3_O_4_ NPs, RGO and Fe_3_O_4_-RGO Nanocomposites

**Figure 5:**
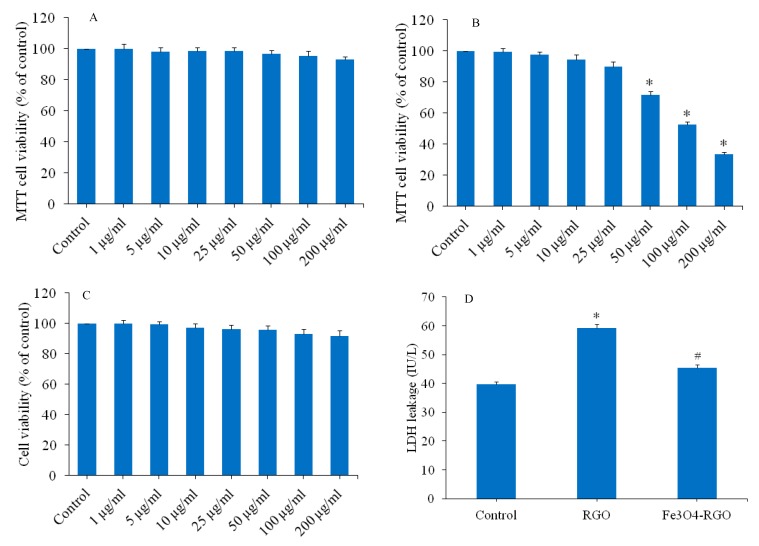
Dose-dependent cytotoxicity of Fe_3_O_4_ NPs, RGO, and Fe_3_O_4_-RGO nanocomposites in HepG2 cells exposed for 24 h. Cell viability against exposure of Fe_3_O_4_ NPs (**A**), RGO (**B**), and Fe_3_O_4_-RGO nanocomposites (**C**). LDH leakage in HepG2 cells after exposure to 100 µg/mL of RGO and Fe_3_O_4_-RGO nanocomposites for 24 h (**D**). Data are represented are mean ± SD of three independent experiments (n = 3). ∗ indicates significant difference from the control (*p* < 0.05). # indicates significant difference from the RGO (*p* < 0.05). RGO: reduced graphene oxide, Fe_3_O_4_: iron oxide, NPs: nanoparticles, LDH; lactate dehydrogenase.

Cells were exposed to different concentration (1–200 µg/mL) of Fe_3_O_4_ NPs, RGO, and Fe_3_O_4_-RGO nanocomposites for 24 h, and cytotoxicity was determined by MTT cell viability assay. Figure 5A demonstrated that the Fe_3_O_4_ NPs did not induce cytotoxicity in HepG2 cells in all selected concentrations. Low cytotoxicity to no cytotoxicity of Fe_3_O_4_ NPs was also reported by other investigators [35,36,37]). However, RGO exposure induced dose-dependent cytotoxicity in HepG2 cells in the concentration range of 50–200 µg/mL (Figure 5B). RGO did not decrease cell viability below the concentration of 50 µg/mL. The cytotoxic potential of the RGO sheets was previously reported in several studies [38,39]. Interestingly, Fe_3_O_4_-RGO nanocomposites were shown not to be cytotoxic to HepG2 cells in the concentration range of 1–200 μg/mL (Figure 5C). These preliminary results suggested Fe_3_O_4_-RGO nanocomposite as promising materials for biomedical applications such as targeted drug delivery.

Based on MTT cell viability data, we have chosen a single concentration (100 µg/mL) of RGO and Fe_3_O_4_-RGO nanocomposites to further explore the cytotoxicity mechanisms of these two nanomaterials. Lactate dehydrogenase (LDH) is a cytosolic enzyme that oxidized lactate into pyruvate. LDH enzyme leakage into the culture media is an indicator of membrane damage. LDH leakage has been used as a marker of cell membrane damage [40]. As we can see in Figure 5D, RGO at a concentration of 100 µg/mL significantly induced LDH leakage as compared to control (*p* < 0.05). However, Fe_3_O_4_-RGO nanocomposites were not able to induce significant amount of LDH leakage in culture media and level was almost close to control group. These results suggested that Fe_3_O_4_-RGO nanocomposites showed good biocompatibility toward HepG2 cells.

### 3.3. Apoptotic Response of RGO and Fe_3_O_4_-RGO Nanocomposites

**Figure 6:**
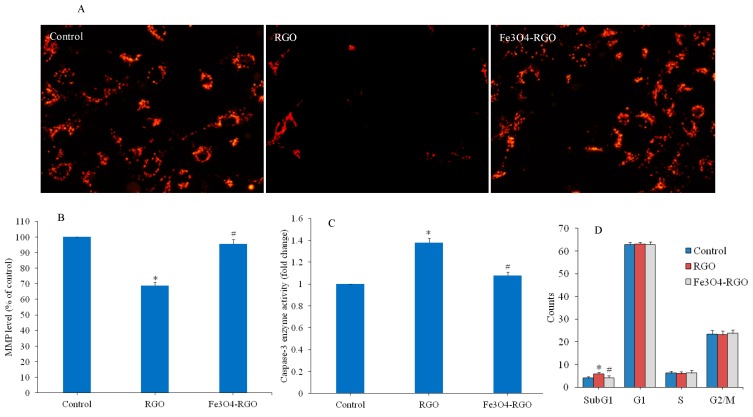
Apoptotic response of HepG2 cells exposed for 24 h to 100 µg/mL of RGO and Fe_3_O_4_-RGO nanocomposites. Fluorescent microscopy images of Rh-123 probe (MMP level) (**A**) and quantitative analysis of MMP level (**B**). Activity of caspase-3 enzyme (**C**) and cell cycle phases (**D**). Quantitative data are represented as mean ± SD of three independent experiments (n = 3). ∗ indicates significant difference from the control (*p* < 0.05). # indicates significant difference from the RGO (*p* < 0.05). MMP: mitochondrial membrane potential, RGO: reduced graphene oxide, Fe_3_O_4_: iron oxide.

Apoptosis is a highly regulated phenomenon of cell death through which tissue get rid of damaged cells [41]. Apoptosis is regulated by various factors such as environmental contaminants, growth factors and deficiency of nutrients [42]. Apoptotic response to GO and RGO is also reported in recent literature [43,44]. Ali et al. [45] observed that Ag-doped RGO induce apoptosis in human liver cells. In this study, we further assessed the apoptotic response of RGO and Fe_3_O_4_-RGO nanocomposites in HepG2 cells. Mitochondria have been shown to play a major role in NPs-induced cytotoxicity [46]. MMP loss is an important incident in deciding cell fate, especially in apoptosis. We examined the MMP level in HepG2 cells after exposure to 100 µg/mL of RGO and Fe_3_O_4_-RGO nanocomposites for 24 h. Fluorescent microscopy data demonstrated that brightness of Rh-123 probe in RGO group was much lower (indicator of MMP loss) than those of control cells (Figure 6A). The fluorescence Rh-123 probe in the Fe_3_O_4_-RGO nanocomposite was similar to the control group. Similar to microscopy data, quantitative results also indicated that MMP level in RGO was significantly lower as compared to control (*p* < 0.05). However, MMP level in Fe_3_O_4_-RGO nanocomposites was significantly higher than the RGO group (*p* < 0.05) and very close to the control group (Figure 6B). We also noted that activity of apoptotic enzyme caspase-3 was significantly higher in the RGO group in comparison to the control group. Again, the activity of caspase-3 enzyme in the Fe_3_O_4_-RGO nanocomposite group was significantly lower than the RGO group (*p* < 0.05) and similar to the control group (Figure 6C).

Cell cycle phases were further analyzed in HepG2 cells after exposure to 100 µg/mL of RGO and Fe_3_O_4_-RGO nanocomposites for 24 h. Cells with damaged DNA accumulated in G1 (gap1), S (DNA synthesis), or in G2/M (gap2/mitosis) phases. However, the cells with damaged DNA are destined to apoptotic cell death and gathered in the subG1 phase [47]. Flow cytometer results showed that RGO induce apoptosis. However, Fe_3_O_4_-RGO nanocomposites did not induce apoptosis in HepG2 cells. Cell gathering in the SubG1 phase of the RGO group was higher (5.86%) compared to the control group (3.98%) (*p* < 0.05) (Figure 6D). Interestingly, cells accumulated in SubG1 phase of the cell cycle in the Fe_3_O_4_-RGO nanocomposite–treated group (4.11%) were similar to the control group (3.98%) (Figure 6D). These results suggested that RGO induce apoptosis in HepG2 cells, whereas Fe_3_O_4_-RGO nanocomposites were not able to cause apoptosis in HepG2 cells.

### 3.4. Oxidative Stress Response of RGO and Fe_3_O_4_-RGO Nanocomposites

**Figure 7:**
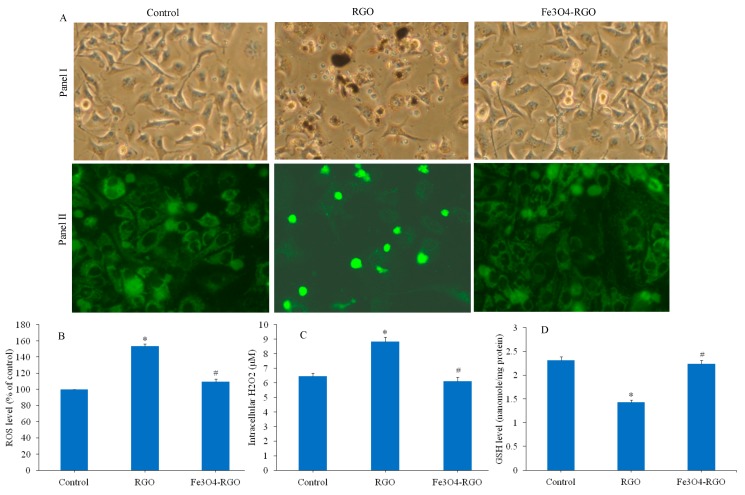
Oxidative stress response of HepG2 cells exposed for 24 h to 100 µg/mL of RGO and Fe_3_O_4_-RGO nanocomposites. (**A**) Panel I represents the phase-contrast microscopic images, and panel II depicts the fluorescent microscopy images of DCF probe (ROS level). (**B**) Quantitative analysis of ROS. (**C**) Intracellular H_2_O_2_ level. (**D**) Antioxidant GSH level. Quantitative data are represented as mean ± SD of three independent experiments (n = 3). ∗ indicates significant difference from the control (*p* < 0.05). # indicates significant difference from the RGO (*p* < 0.05). RGO: reduced graphene oxide, Fe_3_O_4_: iron oxide, ROS: reactive oxygen species, DCF: dichlorofluorescein, H_2_O_2_: hydrogen peroxide, GSH: glutathione.

The high surface area to volume ratio of nano-scale materials leads to higher chemical reactivity causing increased generation of intracellular ROS [48,49]. Oxidative stress arises from an imbalance between ROS generation and their degradation by antioxidants in the cells. Higher production of ROS and oxidative stress is responsible for many ill effects including DNA damage, protein and lipid oxidation, apoptosis, carcinogenesis, and aging [50]. ROS such as superoxide anion (O_2_^•−^), hydroxyl radical (HO^•^), and hydrogen peroxide (H_2_O_2_) serve as signaling molecules in the pathway of apoptosis [51]. Induction of oxidative stress by GO and RGO nanosheets in human cells was earlier reported by several investigators [38,52]. Recently, Shaheen et al. [53] demonstrated that GO-ZnO nanocomposites induce cytotoxicity in human breast cancer (MCF-7) cells through the generation of ROS. Hence, we examined the various biomarkers of oxidative stress in HepG2 cells after exposure to 100 µg/mL of RGO and Fe_3_O_4_-RGO for 24 h. The DCFH-DA probe was used to examine the intracellular level of ROS. Fluorescent images (Figure 7A) depicted that brightness of DCF probe (marker of ROS level) was higher in RGO treated cells than those of control. On the other hand, the brightness of DCF probe in Fe_3_O_4_-RGO nanocomposite—treated cells was similar to that of the control cells. Rounded morphology and detachment of cells from surface after RGO exposure also supported the ROS data. In the control and Fe3O4-RGO nanocomposite groups, cellular morphology was normal. Besides, our quantitative data suggested that the ROS level was significantly higher in the RGO group than those of the control group. Interestingly, ROS level in Fe_3_O_4_-RGO nanocomposites group was significantly lower than RGO group (*p* < 0.05) and nearly close to the control group (Figure 7B). We further examined the intracellular H_2_O_2_ level in HepG2 cells after exposure to RGO and Fe_3_O_4_-RGO nanocomposites for 24 h. Figure 7C demonstrates that the H_2_O_2_ level in RGO group was significantly higher as compared to the control group. Again, the H_2_O_2_ level in the Fe_3_O_4_-RGO nanocomposite group was significantly lower than the RGO group (*p* < 0.05) and close to the control group.

Cells have several antioxidant molecules and enzymes that maintain the redox homeostasis. For example, glutathione (GSH) plays an important role in defense of cells against oxidative damage. Several enzymes such as thiol reductases and peroxidases depend on GSH pool as their source of reducing equivalents [54]. GSH molecule is also associated with either stimulation or induction of apoptosis [55]. We can see in Figure 7D that RGO significantly decreased the GSH level in HepG2 cells as compared to control cells. Interestingly, GSH level in Fe_3_O_4_-RGO nanocomposite group was significantly higher than the RGO group (*p* < 0.05) and very close to the control group. These results suggested that RGO induces ROS generation and oxidative stress in HepG2 cells. However, Fe_3_O_4_-RGO nanocomposites did cause oxidative stress in HepG2 cells. Possible mechanisms through which biocompatible bare Fe_3_O_4_ NPs converted the cytotoxic RGO into biocompatible Fe_3_O_4_-RGO nanocomposites is still remains a future task.

## 4. Conclusions

Prepared Fe_3_O_4_ NPs, RGO, and Fe_3_O_4_-RGO nanocomposites were characterized by UV-visible spectrophotometer, XRD, SEM, TEM, and EDS. Toxicity of RGO and Fe_3_O_4_-RGO nanocomposites were examined in human liver (HepG2) cells. RGO significantly induced cell viability reduction, LDH leakage, MMP loss, and cell cycle arrest. RGO was also found to increase intracellular ROS & H_2_O_2_ levels while decrease the antioxidant GSH level. Interesting results were that Fe_3_O_4_-RGO nanocomposites did not induce cytotoxicity, oxidative stress and apoptosis response in HepG2 cells. Overall, our data demonstrated that Fe_3_O_4_-RGO nanocomposites showed good biocompatibility at cellular level (HepG2). This preliminary study warrants further research for the development of inorganic nanoparticles and graphene-based nanocomposites for biomedical applications.

## Supplementary Materials

The following are available online at https://www.mdpi.com/1996-1944/13/3/660/s1, Figure S1: Chemical composition of prepared Fe_3_O_4_-RGO nanocomposites was determined by energy dispersive spectroscopy (EDS) associated with transmission electron microscopy (TEM). The EDS spectra suggest that Fe, O and C were main elements in Fe_3_O_4_-RGO nanocomposites. The peaks of Cu and C were observed due to use of carbon coated copper TEM grid.
