# Supplementary material for: Investigation of Cytotoxicity, Apoptosis, and Oxidative Stress Response of Fe3O4-RGO Nanocomposites in Human Liver HepG2 cells
Source: Materials (Basel). 2020 Feb 2;13(3):660. doi: 10.3390/ma13030660 (PMC7040707; doi:10.3390/ma13030660)
Supplement: Supplementary file 1 [file materials-13-00660-s001.pdf]

materials

Supplementary Information

Investigation of Cytotoxicity, Apoptosis, and Oxidative Stress Response of Fe_3O_4-RGO Nanocomposites in Human Liver HepG2 cells

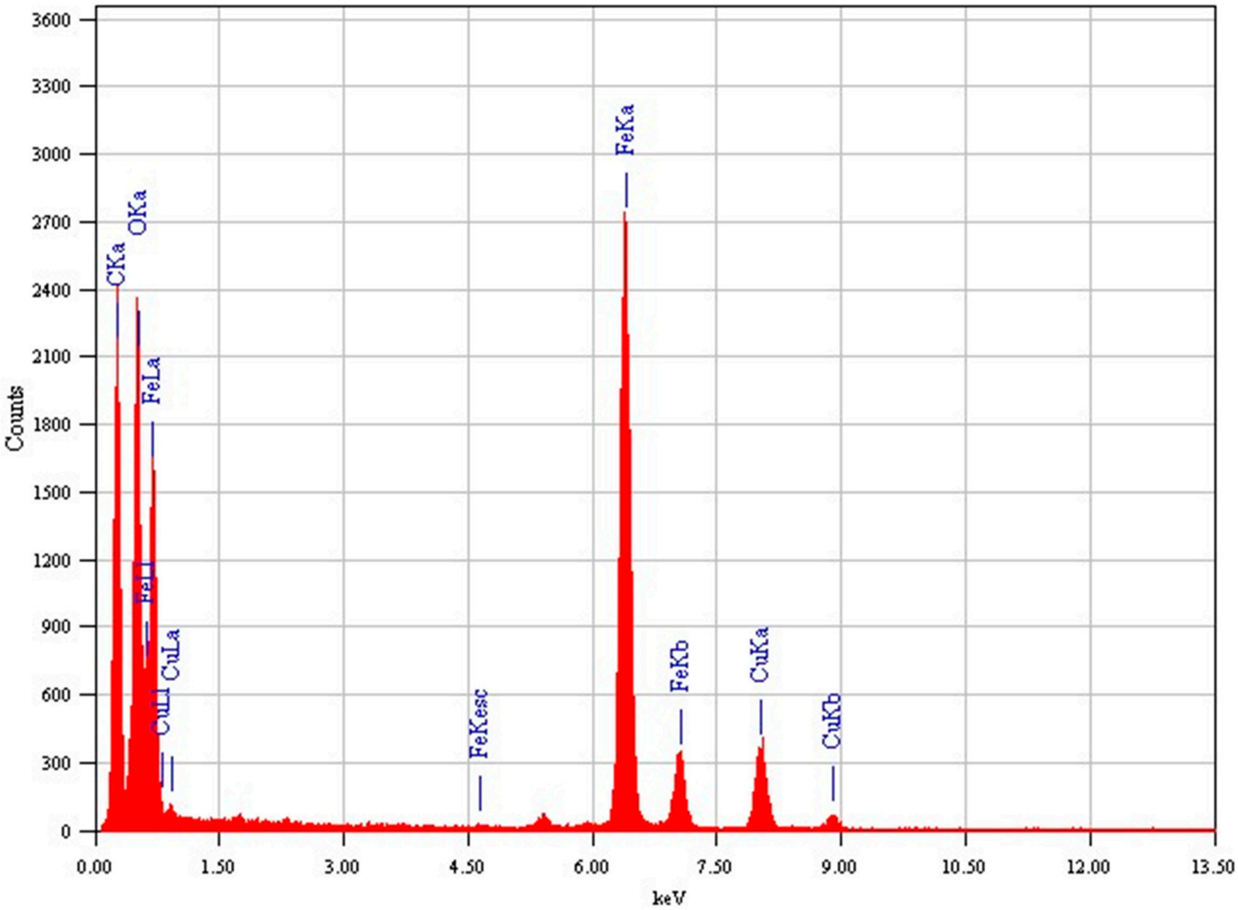

Figure S1. Chemical composition of prepared Fe_3O_4-RGO nanocomposites was determined by energy dispersive spectroscopy (EDS) associated with transmission electron microscopy (TEM). The EDS spectra suggest that Fe, O and C were main elements in Fe_3O_4-RGO nanocomposites. The peaks of Cu and C were observed due to use of carbon coated copper TEM grid.
